# Intelligence, Personality, and the Prediction of Life Outcomes: Borghans et al. (2016) vs. Zisman and Ganzach (2022) Debate

**DOI:** 10.3390/jintelligence11050095

**Published:** 2023-05-15

**Authors:** Lazar Stankov

**Affiliations:** School of Psychology, University of Sydney, Sydney 2006, Australia; lazar.stankov@sydney.edu.au

**Keywords:** intelligence, personality, life outcomes, Big Five

## Abstract

This article examines the psychological measures employed in studies that compared the predictive validity of personality and intelligence for important life outcomes and came to divergent conclusions. At least some discrepant findings can be accounted for by the fine-grained analysis of measures employed in the assessment of intelligence and personality. The use of Big Five measures of personality traits for predicting life outcomes appear to be poorly supported—other ways of assessing personality need to be explored. Methods used to study cause–effect relationships in non-experimental studies will need to be employed in future.

## 1. Introduction

It is common knowledge that both cognitive and non-cognitive psychological processes influence life outcomes (see Ackerman 1996; Stankov 1999). This view was accepted by the participants in a recent debate about the relative roles of intelligence and personality. Borghans et al. (2016) argued that grades and achievement tests are generally better predictors of life outcomes than “pure” measures of intelligence because they capture aspects of personality that are predictive on their own. The abstract of their paper states that “personality is generally more predictive than IQ on a variety of important life outcomes” (p. 13354). Their argument about the prediction was supported by findings from four large datasets. Zisman and Ganzach (2022) re-analyzed two of the same datasets together with four additional ones and came to the opposite conclusion—intelligence is a better predictor of educational (grade point average) and occupational (income) success. In their comments, Golsteyn et al. (2022) pointed out that the discordant findings across the datasets are driven by differences in the measures used, the choice of a particular version of the measure, the populations considered, the circumstances under which tests were taken, and the availability of life outcomes in each dataset. In a reply, Ganzach and Zisman (2022) did not question these points, but the title of their paper emphasizes that “the claim that personality is more important than intelligence in predicting important life outcomes has been greatly exaggerated”. However, neither group of authors attempted to take a closer look at the psychological measures within the different datasets to elaborate on the possible reasons for the discordant conclusions. This commentary is based on a closer look at the data presented by these two groups of authors.

While I welcome the suggestion that psychology can be helpful in predicting life outcomes, I also think that it is unnecessary/premature to argue about the relative roles of intelligence and personality. It is debatable whether intelligence and personality are adequately measured in any of the datasets employed in the two studies. Even though the role of cognitive processes has been amply documented, the question remains as to whether the cognitive tests employed, in fact, measure intelligence properly. The role of personality seems to depend on the way it is defined and measured. The assessment of life outcomes (i.e., the criteria to be predicted) is obviously important, since it is likely that cognitive and non-cognitive predictors will affect them in different ways. Informed discussions about the choice of life outcome variables are needed.

In the following sections, I shall first comment on the usefulness of the psychological measures employed in both studies and point out that at least some discrepant findings can be accounted for by the measures used to assess intelligence and personality, particularly the latter. The last section mentions some of the recent trends in research that have been inspired, in part, by the discussions about the importance of cognitive and non-cognitive processes for life outcomes.

## 2. Comments on Psychological Measures

### 2.1. Cognitive Processes: Partial Measures of Intelligence and the Distinction between Gf and Gc

Only one composite score from both studies may be seen as a proper measure of intelligence. It was used in Borghans et al.’s (2016) study that employed a sample of participants from the NLSY79 dataset. Their intelligence scores were based on five IQ tests, including the Stanford–Binet Intelligence Scale and Wechsler Intelligence Scale for Children. The formation of the composite was not sufficiently described, and its reliability was not reported. Because of their correlations with measures of intelligence, two tests in the Zisman and Ganzach (2022) study—the Henmon–Nelson Test of Mental Abilities and Peabody Picture Vocabulary test—can be considered as proxies for IQ. There are also a couple of tests that are ‘narrow’ (see Gignac 2015) and capture cognitive processes classified under the constructs of fluid intelligence (Gf, Raven’s Progressive Matrices and the matrices test from the British Ability Scale).

In two datasets, the employed measures of intelligence were the Armed Forces Qualifying Test (AFQT) and the average of literacy and numeracy scores from the PIAAC. These tests were seen as measures of achievement, not intelligence, by Golsteyn et al. (2022) who emphasized that achievement tests capture both intelligence and personality. It can be concluded that the measurement of intelligence is not ideal in either of the two studies. Nevertheless, the evidence gathered suggests that proper measures of intelligence may become noteworthy predictors of life outcomes. This is because in the Zisman and Ganzach (2022) paper, the average R^2^ for predicting educational attainment from cognitive tests in six studies is .232 (r = .482), and it is .080 (r = .283) for predicting pay. These two values change little (i.e., to r = .449 and r = .243) if the AFQT and PIAAC studies are excluded from the calculation of the average R^2^. These average correlations can be interpreted as indicating large and medium effect sizes according to Cohen (1992), who suggested that r = .10 should be seen as small, r = .30 medium, and r = .50 as large. In our own work, we used a mid-way point between small and medium (r = .20) as a cut-off for noteworthy correlations (Lee and Stankov 2018).

The debate about the outcomes of the Borghans et al. (2016) and Zisman and Ganzach (2022) studies was also unnecessarily focused on the distinction between Gf and Gc. Proper IQ tests such as the WAIS or Stanford–Binet contain several subtests, some of which measure Gf (subtests requiring adaptation to new situations), while others measure Gc (the result of earlier learning and application of some prior, more fundamental general ability such as Gf, Cattell 1963, p. 3). The tests of verbal analogies, vocabulary, synonyms/antonyms, and general information have been used as markers of Gc. These tests differ from the typical achievement tests in, say, mathematics and science. Psychologists view Gc tests as proper measures of intelligence and emphasize the role of fundamental general ability both in prior learning and in concurrent assessments. Borghans et al.’s (2016) assumption that they are akin to achievement tests and therefore related to personality is not widely shared in the field.

As pointed out by Horn (1968), Gc indicates the extent of acculturation as it determines human abilities, and Gf indicates a pattern of neural physiological and incidental learning influences. They show distinct developmental trends over the lifespan, with Gf starting to decline around the mid- to late-twenties and Gc remaining at an even level until the sixties. These distinctions were not acknowledged in any of the studies cited by the two groups of investigators, and yet they have important implications for the interpretation of the relationship between cognition and life outcomes. For example, relative levels of Gf and Gc during the middle of the lifespan may be critical for the development of crucial aspects of life outcomes for the rest of one’s life.

The theory of fluid and crystallized intelligence has evolved over the years and is now known as the Cattell–Horn–Carroll theory (CHC, Schneider and McGrew 2018). Gf and Gc are two among eleven broad abilities that are nowadays seen to comprise intelligence. Talking about intelligence without considering broad factors additional to these two is hard to justify. For example, adding broad abilities of long-term storage and retrieval (Glr, the ability to store information and fluently retrieve it later in the process of thinking) or working memory (Gwm) to the intelligence measure used in any of the above-mentioned large-scale studies may modify the conclusions about the predictive roles of intelligence and personality. It can be noted that this conceptualization also does not imply that learning is necessarily related to personality, as assumed by Borghans et al. (2016), since, in fact, some other cognitive ability—e.g., Glr, Gwm, or, say, processing speed (Gs)—that was not appropriately controlled in their reported studies may affect learning as well. This possibility needs to be explored before making strong claims about the role of personality. Additionally, this implies that without additional evidence, the Gc tests should not be labeled as inappropriate measures of intelligence.

### 2.2. Non-Cognitive Processes: Big Five Personality and Beyond

Conceptual issues related to personality arise from its definition and the inadequate measurement of this domain. Zisman and Ganzach (2022) restrict themselves to the use of datasets with scores from the Big Five measures identified in individual differences studies that employed lexical analysis of natural language in constructing questionnaire items for the assessment of extraversion, agreeableness, conscientiousness, openness, and emotional stability. In their paper (see Table 3, columns 2 and 8), the average R^2^ from six studies is .053 (r = .230) for predicting educational attainment from the Big Five, and it is .040 (r = .200) for predicting pay, both being about half-way between small and medium according to Cohen’s (1992) criterion. Out of the six datasets, Zisman and Ganzach (2022, Table 3) report that only one (WLS dataset) produced a value (R^2^ = .079, r = .281) that is above the cut-off for predicting educational attainment and occupational success (log pay) with personality assessed via the Big Five. This appears to be due mostly to the somewhat higher raw correlations (r = .271 and r = .244) that openness has with these two criteria.

Borghans et al. (2016) also report the findings with the Big Five. The results from one dataset (Stella Maris, Figure 1) broadly agree with the above findings: r = .259 with educational attainment. The second dataset (MIDUS) was analyzed by both groups of investigators, and the results of the analyses, using the log-wage as a criterion, were completely different, with Borghans et al.’s (2016, Figure 6) favoring personality and Zisman and Ganzach’s (2022, Table 3) favoring ability. In an attempt to understand the difference between the results, Zisman and Ganzach contacted Borghans several times but received no response. For that reason, MIDUS data will not be discussed further in this commentary.

In addition, Borghans et al. (2016) reported the results based on personality measures of grit, self-esteem, the locus of control provided by the participants, and teachers’ assessment of disorderly activity, antisocial behavior, introversion, and neuroticism. These measures arose from studies that did not employ a lexical approach but relied on psychological analyses and examinations of aberrant behavior. The distinction between the Big Five personality traits and those of aberrant behavior should not be taken lightly. They may impact on job performance and therefore on life outcomes differently with, for example, the Big Five being closer to performance for some specific jobs and aberrant behavior having broader effects.

Stankov (2018) also argued for the inclusion of measures of personality other than those captured by the Big Five in the prediction of behavior because of the findings that some personality-like dispositions—e.g., dark traits such as Eysenck’s psychoticism and self-beliefs such as self-efficacy—are predictors of both academic and job performance. These are ‘normal’ personality dispositions that can influence life outcomes but are also not captured by the Big Five scales.

### 2.3. Measures of Life Outcomes including Death

In Zisman and Ganzach’s (2022) paper, only two measures of life outcomes were employed: educational success (grade point average and the highest level of education) and income (log pay). In addition to these two, Borghans et al. (2016) used a variety of other measures including life satisfaction, number of arrests, voting, and health-related indices (body mass index, depression, both physical and mental health). These additional indices are obviously related to at least some personality measures of aberrant behavior mentioned above.

Although the selection of life outcome indices in the two papers appears haphazard, the predictive validities reported in both studies strongly suggest the need to develop theoretical frameworks and invest serious research efforts to expand and improve their assessment. Methodological studies in sociology use the term “estimand” rather than criterion (i.e., life outcome), and Lundberg et al. (2021) elaborated on the issues related to its selection and interpretation in a way that may be useful to psychologists and economists.

Decisions will have to be made about the age at which life outcomes should be assessed. Given that much of the research in psychology has focused on younger ages, new indices may have to be developed if midlife or retirement age is to be chosen. A useful measure was suggested by the study of the ultimate validity of psychological tests reported by O’Toole and Stankov (1992). The two groups of participants for this study were selected from a cohort (N = 46,166) of Australian Army National Servicemen who were at least 18 years old when first enlisted between 1 June 1965 and 28 February 1971. The first group were those who were registered as being deceased (N = 523) within 16.5 years (i.e., between June 1965 and 1 January1982) of their enlistment. Their ages at death ranged between 22 and 40. The second group were survivors (N = 1786) randomly selected from the same cohort. At the time of enlistment, three cognitive tests were given to all National Servicemen. One was a measure of intelligence, The Army General Classification Test (AGC) that consists of items measuring both Gf and Gc, and the other two were measures of perceptual/clerical speed and mechanical knowledge. The finding was that the average scores of survivors were significantly higher than those of the deceased persons on all three tests.

O’Toole and Stankov (1992) took a further step in their analyses and addressed the issue of different predictive roles of personality and intelligence. Two major causes of death were identified: (a.) external, i.e., motor vehicle accidents (MVA, N = 314), and (b.) internal, i.e., suicides (N = 76). Using logistic regression, it was again found that both MVA and suicides were well-predicted by the AGC test scores—those with low intelligence scores were more likely to experience death than those with high scores. In addition, those who committed suicide also had significantly higher AWOL (absent without official leave) incidents and more psychiatric problems, suggesting the (negative) role of personality. For the MVA group, additional but somewhat weak predictors were criminal record and level of education.

Overall, measures of survival (i.e., death rates) before the age of 40 (or perhaps the end of the midlife) can be added to the list of important life outcomes in studies of the predictive validity of psychological variables.

Stankov (1999) pointed out that the predictive validity of cognitive abilities for life achievements/outcomes become non-significant, i.e., intelligence assessed in childhood may have a low correlation with criterion variables at the old age, implying that non-cognitive processes may be better predictors. A meta-analysis carried out by Sanchez-Izquierdo et al. (2023) used survival (age of death) as a criterion and arrived at the same conclusion. They pointed out that the positive relationship between intelligence and survival is dampened with age. Intelligence acts as a protective life outcome factor prior to reaching upper-middle age, but survival in old-age depends more on social and health behaviors during the lifespan.

## 3. Need to Improve the Assessment of Personality in Studies of Life Outcomes

### 3.1. Big Five Personality Scales: Poor Predictors of Income and Educational Attainment

For psychologists interested in individual differences in cognitive abilities and personality, the findings summarized above are not surprising. Educational achievement is well-known to be related to intelligence, and although income/pay has not often been used as a criterion, the reported correlations do make sense. The suggestion that personality is generally more predictive than IQ is an exaggeration, at least with respect to the two outcomes considered in both papers. Nevertheless, as mentioned above, personality defined in terms of the Big Five is correlated about mid-way (r = .230 and r = .200) between the small and medium effect sizes and therefore may be claimed to be not negligible. These estimates were based on multiple correlations between the life outcomes and five component scores of the Big Five. The average correlations between life outcome measures and scores from each of the Big Five components in the six studies tell a different story. These are presented in the first two rows of Table 1.

It is evident that all correlations but one, i.e., openness with educational attainment, are smaller than r = .10. In our own work, Openness and sometimes Conscientiousness were the only components of the Big Five that occasionally had noteworthy correlations (i.e., close to r = .30) with measures of academic performance and intelligence (Stankov 2018). For comparative purposes, the last row presents correlations between the general intelligence ‘g’ and the Big Five components as reported by Anglim et al. (2022). Clearly, virtually all components of the Big Five have negligible correlations with the two measures of life outcomes and intelligence (i.e., g). The difference between predictive validity based on multiple correlations and predictions based on component scores touches upon conceptual issues somewhat unique to psychology. Multiple correlations’ coefficient R captures not only each component’s contribution to the criterion but also correlations between the components. This raises questions about the size of correlations between the five components and whether there is a general personality factor analogous to intelligence underlying the Big Five. Although there are claims for the existence of a general personality factor, it is commonly accepted that Big Five components are independent traits. This interpretation would de-emphasize findings with multiple correlations and support the conclusion about the negligible predictability of the Big Five.

A recent paper by Hübner et al. (2022) also argued for the fine-grained analysis of the relationship between the Big Five measures and achievement scores based on almost 15,000 students from upper secondary schools in Germany. The sizes of the correlations between the grades and standardized achievement scores in language and mathematics with each Big Five component were comparable to those presented in Table 1. However, they varied depending on the type of assessment employed. Thus, conscientiousness had higher correlations with standardized achievement scores of language tests (i.e., measures that did not have strong ‘cognitive ability saturation’), and openness had higher correlations with mathematics grades (i.e., non-standardized tests that did require strong cognitive ability). It remains to be seen whether attempts to use fine-grained analyses of the important life outcome criterion variables will also lead to a substantial increase in the effect sizes.

### 3.2. The Need to Broaden the Domain of Personality

As mentioned above, personality is a broad term, and there are aspects of it that are not captured by the Big Five. What is the evidence for their predictive validity? Two datasets from Borghans et al. (2016) contain relevant information. For the NLSY79 (Figures 3 and 5) dataset that used personality assessments based on measures of locus of control and self-esteem as predictors, measures of educational achievement (AFQT) had an R^2^ = .173 (r = .416) and grades had an R^2^ = .093 (r = .305). However, several life outcome criteria (see Figure 5) did not pass the r = .200 cut-off point. For another dataset (BCS, Figures 2 and 4), personality did not exceed the cut-off point for predicting achievement and wages, but it did exceed the cut-off for predicting education (years in which a degree was obtained: R^2^ = .143 and r = .378).

Overall, the predictive validity of personality measures in both studies is higher than what was obtained with the Big Five, but the evidence is not strong. Unsurprisingly, the level of predictability depends on the choice of the measures of personality and on the predictive criteria employed.

### 3.3. Science vs. Business Is a Tricky Line to Walk

Over the past couple of decades, there has been increased interest in the study of non-cognitive processes and their role in life. In part, this was stimulated by behavioral economists who have become disenchanted with the prevailing emphasis on the general factor ‘g’ of intelligence. In consequence, many research projects that focused on personality were undertaken^1^. 

Large-scale studies of what is now referred to as Social and Emotional Skills are currently supported by the OECD and its Programme for International Student Assessment (PISA, see Kankaraš and Suarez-Alvarez 2019; OECD 2021). This is welcome encouragement for psychological research. However, the debate between Borghans et al. (2016) and Zisman and Ganzach (2022) suggests that the approach adopted by the PISA may not be optimal. In particular, the PISA team placed strong emphasis on the Big Five model, which has been used as a framework for the study of non-cognitive social and emotional skills. The same framework has been adopted by some commercial assessment companies. Although this model may have value for predicting some individual and societal criteria (Soto 2021), it has limited value for predicting the life outcome criteria of academic and occupational success of interest to behavioral economists.

In recent studies, the focus has shifted from the Big Five domains to their narrower sub-components called ‘facets’ and item-level ‘nuances’ that are expected to have higher predictive validity. Some interpret facets as analogous to the primary factors of the Gf, Gc, and other lower-order components of the Gf/Gc theory of intelligence. While this similarity may improve with further work in future, at present, each facet appears to be a subjective selection of several items that are presumed to measure an aspect of the domain. There seems to be no agreement about the standard set of facets that define a domain. For example, in a PISA pilot study, the open-mindedness (openness to experience) domain included the facets of curiosity, tolerance, and creativity (OECD 2021). However, Rouco et al. (2022) listed the following nine facets of openness: creativity, adventurousness, open-mindedness, interest in reading, culture, curiosity, willingness to learn, empathy, and intellect. Currently, there is no empirical evidence showing a relationship between the different facets of, presumably, the same domain, and there is little evidence of the facets’ predictive superiority over the broader domains of the Big Five.

There are ways other than the use of the Big Five framework that can help to expand the list of non-cognitive processes predictive of important life outcomes. One of these involves searching the literature for the strong predictors among the well-established non-cognitive psychological and social measures. This thought motivated Lee and Stankov’s (2018) study that reported correlations between the PISA and TIMSS mathematics achievement scores and a host of non-cognitive variables gathered in these two large-scale international programs. Two psychological variables stood out as good predictors of achievement. One of these was self-efficacy (example item: “How confident do you feel about … calculating the number of square meters of tile you need to cover a floor”), which correlated r = .461 with PISA 2012 mathematics achievement. The other was mathematics test anxiety which had a correlation of r = −.365. These two constructs are likely to be good predictors of life outcomes related to educational success. Stankov (2018) also pointed out that a measure of confidence has an even higher (r = .555) correlation with performance. It may pay off to search for other strong non-cognitive predictors instead of using the untested facets of the Big Five. A step in this direction has already been taken in the PISA study of Social and Emotional Skills. Self-efficacy was included as a “compound skill”, i.e., additional to the facets of the Big Five that do not capture it (OECD 2021).

### 3.4. Methodological Issues

The debate about the relative roles of personality and intelligence in relation to important life outcomes touches upon broader issues related to scientific methodology and theory in psychology. Out of the many issues that may be addressed under this topic, I shall briefly mention the following three: (a.) the need for longitudinal, multi-stage studies; (b.) the need to control measurement errors in large-scale studies; and (c.) the need to design non-experimental studies that point to causal relationships.

Longitudinal studies of important life outcomes, perhaps with regular assessments every decade over the whole life span, are missing at present. More common are studies with school-age children, and two of Bardach et al.’s (2023) findings are relevant to our discussion. They assessed intelligence, personality (Big Five), and school achievement in mathematics four times between the ages of 11 and 14 (i.e., in grades 5, 6, 7, and 8). First, they report that personality is not significantly related to academic achievement, diminishing Borghans et al.’s (2016) argument that achievement scores can be decomposed into intelligence and personality. Second, they found that academic achievement in mathematics and intelligence have “reciprocal within-person relations, with the strongest coefficients found for achievement longitudinally predicting intelligence” (abstract). In other words, higher scores in mathematics at a younger age are associated with higher intelligence scores in subsequent years, suggesting that educational interventions may be effective in increasing IQ scores. One may wonder if similar findings will be obtained with the older age groups, e.g., whether engagement in some particular cognitively demanding activities during the lifespan leads to a slowing in Gf decline and better life outcomes.

One reason for the discrepant findings between the two groups of investigators may be related to the size of the measurement error (see van Smeden et al. 2020). In typical studies of individual differences in psychology, the reliability of the measures is reported, and neither group of investigators used instruments that had unacceptably low reliability. Furthermore, I was personally assured by one of the authors that they were very careful to use the same databases, the same measure, and the same method of analysis. Nevertheless, some discrepancies might still be due to the presence of measurement errors and inappropriate control, which should be considered in future work.

Both reviewers of the present paper pointed out that in non-experimental studies, the emphasis has been on the prediction rather than on causal effects. Grosz et al. (2020) argued that approximation to causal relationships can be inferred from the non-experimental studies by using, for example, directed acyclic graphs (DAGs). These can be understood in terms of the structural equation models (SEMs) that are commonly used in psychology. A set of variables related to a given outcome forms a DAG if all lines connecting them are one-directional rather than two-directional (i.e., indicating covariance) and if they do not form a closed loop (acyclic). In a sense, the reviewers argued for the replacement of correlations and regression analyses with SEMs/DAGs embedded with causal psychological interpretations, as also suggested by Grosz et al. (2020). In my own opinion, this approach will need to be used in future studies. I also believe that findings that focus on prediction are useful and cannot be dismissed offhand.

## 4. Conclusions

There can be no doubts that cognitive and non-cognitive psychological variables can predict important life outcomes such as income, academic achievement, and aspects of aberrant behavior that were included in the reports of Borghans et al. (2016) and Zisman and Ganzach (2022). It remains to be seen whether proper tests of intelligence can also do the job. As for personality, the verdict from the two reports depends on its definition, with the Big Five having both inferior predictability in comparison to cognitive measures and a low effect size (i.e., correlations with the life outcome criteria).

Most future work will need to be carried out with respect to non-cognitive processes. Personality will need to include a broader set of non-cognitive dispositions. The list of life outcomes also needs to be expanded beyond those considered in the reports, with age and causes of death being a possible addition. Many methodological issues will need to be addressed in the design and execution of future studies. Clearly, there is plenty of work ahead but, without doubt, this will be a worthwhile undertaking.

## Figures and Tables

**Table 1:** Average Correlations between the Big Five and the Intelligence and Life Outcomes (see Zisman and Ganzach’s (2022) Appendix A.7) and Anglim et al. (2022).

Life Outcomes	Big Five					Multiple Correlations ^#^
	Conscientiousness	Extraversion	Agreeableness	Emotional Stability	Openness	
Educational Attainment	.074	.028	.008	.049	.148	.232
Log Pay	.069	.011	−.062	.069	.073	.200
Anglim et al. (2022) g	−.020	−.010	.000	−.080	.170	

^#^ Calculated as a square root of the average of personality columns (2 and 8) in Table 3.

## Data Availability

Not applicable.

## Note

1 The title of Section 3.3 (“Science vs. Business Is a Tricky Line to Walk”) is the phrase that was used by one of my colleagues after prolonged discussions about the issues raised in this commentary. The person was engaged in developing new personality measures for the prediction of life outcomes and has relied heavily on the Big Five model in this endeavor. Apparently, research support is easier to get if your proposal is linked to the Big Five.
